# When the chimney is blocked: malignant renovascular hypertension after endovascular repair of abdominal aortic aneurysm

**DOI:** 10.1186/1471-2369-14-71

**Published:** 2013-03-26

**Authors:** Amir Gal-Oz, Yehuda G Wolf, Galia Rosen, Haggai Sharon, Idit F Schwartz, Gil Chernin

**Affiliations:** 1 Department of Nephrology, Tel-Aviv Sourasky Medical Center and Sackler Faculty of Medicine, Tel-Aviv University, Tel-Aviv, Israel; 2 Department of Vascular Surgery, Tel-Aviv Sourasky Medical Center and Sackler Faculty of Medicine, Tel-Aviv University, Tel-Aviv, Israel; 3 Department of Radiology, Tel-Aviv Sourasky Medical Center and Sackler Faculty of Medicine, Tel-Aviv University, Tel-Aviv, Israel; 4 Nephrology Department, Tel- Aviv Sourasky Medical Center, 6, Weizmann Street, Tel-Aviv 64239, Israel

**Keywords:** Abdominal aortic aneurysm (AAA), Chimney graft (CG), Endovascular Aneurysm Repair (EVAR), Renovascular hypertension

## Abstract

**Background:**

The Chimney graft (CG) procedure is one of the novel modification techniques of the endovascular aneurysm repair (EVAR) surgery to treat suprarenal and juxtarenal abdominal aortic aneurysms. Other indications for the use of CG placement include thoracic and thoracoabdominal aneurysms with supraortic branches orifice involvement and cases of common iliac artery aneurysms with or without internal iliac artery involvement. The technique is used in patients who due to aortic-neck morphology and lack of adequate fixation and/or sealing zones are not eligible for standard EVAR. In this procedure, a parallel stent-graft is placed adjacent to the main body of the aortic endograft to maintain blood supply to renovisceral or supraortic branches, once the body of the aortic stent-graft is deployed. Symptomatic occlusions of the CG with novel renovascular hypertension were not described until now.

**Case presentation:**

A-64-year-old male patient, presented with new-onset malignant hypertension, 13 months after an EVAR operation with CG placement to the left renal artery. The patient was on preventive clopidrogel therapy, which was withheld temporarily for several days, one month before presentation. Imaging studies revealed a novel form of iatrogenic renovascular hypertension, caused by occlusion of the CG. Any attempt to recanalize the covered stent or revascularize the left kidney was rejected and conservative treatment was chosen. Seven months after presentation, blood pressure was within normal ranges with little need for antihypertensive therapy.

**Conclusions:**

Physicians should be aware that the novel emerging techniques of EVAR to overcome the limitations of the aortic-neck anatomy may still adversely influence the renal outcome with potential development of new-onset hypertension.

## Background

Endovascular aneurysm repair (EVAR) has gained widespread acceptance as the procedure of choice for repair of infrarenal abdominal aortic aneurysms (AAA) in patients with suitable aortic anatomy [1]. The improvement of the technique and surgeon’s skills has extended the possible indication for repair to patients with a more complex aortic anatomy [1,2]. Despite these improvements, the main anatomic limitation to EVAR is the proximal aortic-neck anatomy. There is no consensus about the method of choice to repair suprarenal or juxtarenal AAAs [2]. Open repair is an effective method, yet the post-operative risks and the high morbidity and mortality rates make this operation suitable to low-risk patients [3]. The standard EVAR procedure for infrarenal AAAs cannot be applied to suprarenal or juxtarenal AAAs since it is accompanied by high rate of adverse events including proximal endoleak, migration and mainly aortic side branches occlusion with possible renovisceral ischemia [1,2,4]. Novel modifications of the traditional EVAR procedure have evolved in order to offer less invasive approach to repair suprarenal or juxtarenal AAA. The fenestrated and branched EVAR techniques have gained popularity as a possible alternative to open repair or standard EVAR techniques that would not compromise blood perfusion to the aortic side branches, since the sealing and/or fixation zone is translocated proximal or distal away from the target vessels [4,5]. However, the endovascular stent grafts that are used in the fenestrated and branched EVAR are custom-made and require measuring, fitting and a period of time for graft preparation and supply. Therefore, these two costly techniques are only suitable for elective repair of AAA and not in emergent cases [1,2,4,5]. In 2003, Greenberg *et al*, have introduced the novel chimney graft (CG) procedure (a.k.a. the “snorkel technique”) in order to overcome some of the limitations of the fenestrated and branched EVAR [6]. The CG technique involves placing of parallel stent grafts adjacent to the main body of the aortic endograft to maintain blood supply to renal and other visceral branches post- aneurysm exclusion. Contrary to EVAR with the fenestrated or branched technique, the CG technique involves standard off-the-shelf stent grafts and can be used in emergent cases [1,2]. Possible indications for the use of CGs, other than treating suprarenal and juxtarenal abdominal aortic aneurysms, include thoracic and thoracoabdominal aneurysms with supraortic branches orifice involvement and suitable cases of common iliac artery aneurysms with or without internal iliac artery involvement [7,8]. A potential major drawback of the EVAR procedures is the presence of endoleaks (*i*.*e.* persistent flow of blood into the aneurysm sac after device placement). Endoleaks were reported to complicate up to 25% of EVAR operations with CG placement. However, endoleak repair not necessary in most cases [1].

Hereby we present a novel etiology of malignant renovascular hypertension caused by a renal artery CG occlusion.

## Case presentation

**Figure 1:**
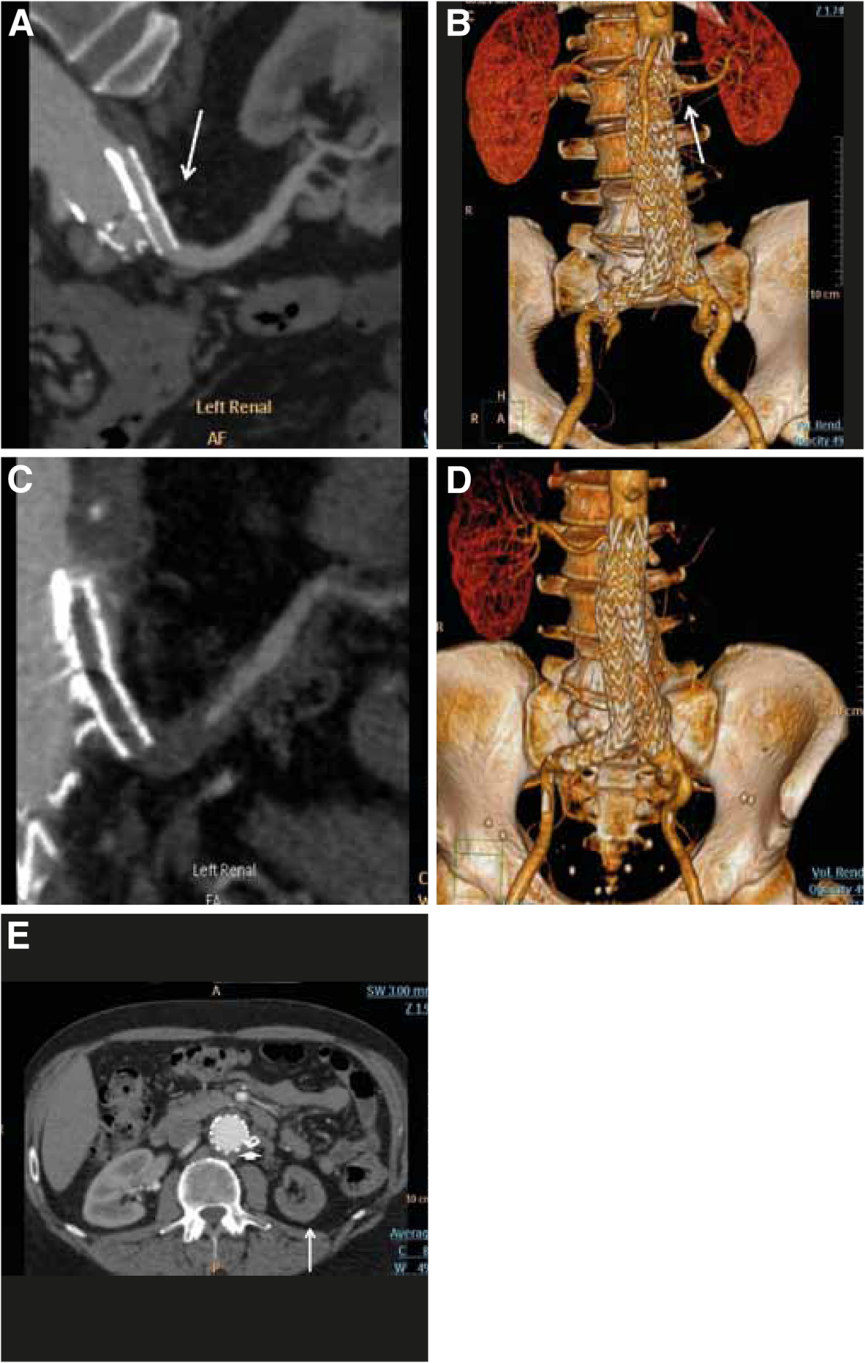
**Abdominal CT-angiography (CTA) of a 64-year old patient after EVAR with placement of GC into the left renal artery. A-B.** Five months before presentation, during routine post-operative follow up and **C-E.** At presentation with malignant renovascular hypertension; **A.** Curved Multi Planar Reconstruction (cMPR) image showing a patent CG (arrow). **B.** A volume rendering image showing patent aortic stent-graft and patent CG (arrow) with symmetric enhancement of the two kidneys. **C.** cMPR image showing occluded CG with near complete occlusion of the left renal artery. A trickle of contrast is demonstrated in a distal segment of the artery. **D.** A volume rendering image showing the occluded CG with lack of enhancement of the left kidney. **E**. Axial image showing the occluded CG (short arrow) and small, shrunken non enhancing left kidney (long arrow).

A 64-year-old male patient was admitted to the emergency ward for severe occipital headache, visual disturbances and new-onset severe hypertension. The symptoms were initially noted three days before admission. His medical history was notable for hypercholesterolemia with atorvastatin therapy. Thirteen months before admission, an EVAR operation was performed to repair a rapidly expanding AAA with a diameter of 53 mm. EVAR with Endurant endograft (Medtronic, Minneapolis, MN) was combined with a Chimney procedure into the left renal artery, using a balloon expandable covered stent-graft (7 × 38 mm Advanta V12; Atrium Medical Corp). The Chimney technique was used due to irregular aortic neck with posterior ulceration and high difference of right renal artery origin, 10 mm above the left renal artery origin. Antiplatelet therapy with clopidrogel (75 mg/day) was initiated after the surgery. During the 6th month follow-up an abdominal computed-tomography angiography (CTA) revealed a well functioning aortic stent and a patent CG (Figure 1A-B). One month before presentation with hypertension, clopidrogel therapy was temporarily stopped for six days, in order to undergo an elective laparoscopic bilateral inguinal hernia repair (IHR) surgery. The immediate-post operative follow-up after the IHR was uneventful with normal blood pressure measuring.

At admission, the patient was without abdominal or flank pain. Blood pressure was 224/113 mmHg, heart rate 91 beats per minute. Ophthalmology examination including optical coherence tomography (OCT) revealed retinal detachment and a large accumulation of subretinal fluid in the right eye. There were no murmurs or bruits over the renal arteries. Laboratory results at admission with comparison to values taken five weeks before admission are summarized in Table 1.

**Table 1:** Laboratory results at admission and five weeks before admission

**Parameters**	**At admission**	**Five weeks before admission**
**Serum creatinine (mg/dl)**	1.5	1.2
**Potassium (meq/L)**	3.8	4.2
**Sodium (meq/L)**	138	140
**Lactate dehydrogenase (U/L)**	436	288
**Alkaline phosphatase (U/L)**	114	79
**Bicarbonate meq/L**	24	-
**Hemoglobin g/dl**	12.1	10.1
**WBC, (x10**^**3**^**/μl)**	8.7	7.1
**Platelets (x10**^**9**^**/L)**	180000	152000
**Plasma renin activity (PRA) in the supine position (ng/ml/hr)**	>37	-
**Urinalysis**	Without hematuria or leukocyturia	-

Renal duplex ultrasound failed to detect blood flow into the left renal artery. CTA demonstrated an abdominal endograft that was patent without a leak, dissection or migration. However, a total occlusion of the CG to the left renal artery was noted without stent kinking (Figure 1C-D). The left renal parenchyma was smaller compared to the previous CTA, with decreased contrast media uptake and hypodense wedge areas, indicative of multiple infract (Figure 1E).

Medical therapy to reduce blood pressure was initiated with intravenous nitroglycerine and subsequently with maximal dosage of oral ramipril, amlodipine and hydrochlorothiazide. Blood pressure was lowered gradually and the patient was discharged with improvement of ophthalmological findings and serum creatinine levels of 1.5 mg/dL. Eight months after discharge, the patient was asymptomatic with only ramipril 2.5 mg/day as antihypertensive medication. At that point of time, office blood pressure was measured 128/81 mmHg and serum creatinine levels were of 1.3 mg/dL. The protein excretion in urine was of 280 mg/day.

## Conclusions

The newer techniques of EVAR, such as the fenestrated, branched or CG techniques, can pose an increased risk of renal injury because of several mechanisms, including higher doses of nephrotoxic contrast media and instrumentation of the renal arteries, which can lead to emboli and inadequate revascularization. Other potential causes renal injury are mechanical stenoses or kinking and in-stent restenoses due to neo intimal hyperplasia [5,9]. Indeed, occlusion of renal arteries and decline of estimated glomerular filtration rate (eGFR) were reported with the fenestrated and branched techniques [9]. Several reports have addressed the short and mid-term outcome of EVAR with the use of the CG technique including the renal outcome [10-13]. It is important to remember that intentional coverage of one of the renal arteries is sometime considered in cases with the need of CG into the superior mesenteric artery because of the risk that placing more than two CGs might compromise the proximal seal [11]. A recent review by Moulkakis *et al*. summarized the results of 15 studies with the placement of a total 134 CGs and a mean follow-up of nine months. The patency-rate of CG was 97.8% (131/134) with one patient reported to have renal GC occlusion after 45 days, necessitating urgent thrombectomy and bypass [10]. Another report of 28 patients after EVAR with placement of 56 CGs, found one case of renal CG occlusion after 90 days (98.2% GCs patency). The occlusion was asymptomatic and without deterioration of kidney function [12]. Other adverse renal effects that were described with the CG technique include: dissection of the stented renal artery [13], perinephric hematoma [1], and acute kidney injury (AKI) with the need for renal replacement therapy [13]. A comparison of one-year follow-up of renal outcome in 21 patients treated with EVAR and CG technique *versus* 21 anatomically-matched patients that underwent an open repair surgery found similar decline of eGFR in both groups. Overall, six patients (29%) of the 21 patients treated with EVAR and CG had AKI [11].

To the best of our knowledge, this is the first detailed description of a late occurrence (more than a year post CG placement) of thrombosis to the CG with the presentation of severe renovascular hypertension. The pathophysiology of renovascular hypertension, involves increased activation of the renin-angiotensin axis. Therefore, medical treatment was initiated and maintained with the angiotensin converting enzyme inhibitor, ramipril [14].

Any attempt to recanalize the covered stent or revascularize the left kidney was rejected. The rational for our conservative approach was that the symptoms of malignant hypertension persisted for several days before presentation and that the left kidney was found to be small. We believe that revascularization of occluded renal artery CG, should be considered in acute events without signs of prolonged ischemia to the affected kidney [15]. Different invasive approaches of revascularization are possible, such as percutaneous endovascular therapy or distal bypass. There is lack of sufficient data to indicate which method of revascularization is preferable.

In the case described here, clopidrogel therapy was stopped for several days, one month prior to presentation. It is possible that the temporary perioperative cessation of antiplatelet therapy has initiated, or at least exacerbated, a thrombotic occlusion process inside the CG. The lack of flank pain, the slight elevation of lactate dehydrogenase from baseline measures and the normal urinalysis, may suggest that the thrombosis in the CG was not merely an acute event, just shortly before the appearance of symptoms. There is lack of evidence-based guidelines about the appropriate perioperative antiplatelet therapy for CGs, as well as other stents, after more than a year from stent placement. A balance between the risk of stent thrombosis and the risk of bleeding is hard to find in clinical practice [16]. Our case demonstrates that late-thrombosis, more than a year after placement of a CG, is a potential threat. One needs to consider the merits *versus* the risks of withholding antiplatelet therapy in patients after EVAR with CG, even as a temporary measure before invasive procedures. We believe that a reasonable approach before elective operations for patients with EVAR and CG, is to stop antiplatelet therapy, one week prior to elective surgery and to temporarily initiate anticoagulation treatment (e.g. subcutaneous Enoxaparin). Anticoagulation will be stopped, one day before the procedure, and antiplatelet therapy will be re-initiated in the second post-operative day.

## Consent

The patient has given his consent for the Case reports to be published.

## Competing interests

The authors declare that they have no competing interests.

## Authors’ contributions

AG- participated in the clinical follow-up of the patient and preparation of the manuscript. YGF- participated as the surgeon and in the clinical follow-up of the patient. GR-participated as the radiology consultant and follow-up of the patient. HS- participated in the clinical follow-up of the patient. IFS- participated in the clinical follow-up of the patient; GC- participated in the clinical follow-up of the patient and carried out the final preparation of the manuscript. All authors read and approved the final manuscript.

## Pre-publication history

The pre-publication history for this paper can be accessed here:

http://www.biomedcentral.com/1471-2369/14/71/prepub
